# CMISG1701: a multicenter prospective randomized phase III clinical trial comparing neoadjuvant chemoradiotherapy to neoadjuvant chemotherapy followed by minimally invasive esophagectomy in patients with locally advanced resectable esophageal squamous cell carcinoma (cT_3-4a_N_0-1_M_0_) (NCT03001596)

**DOI:** 10.1186/s12885-017-3446-7

**Published:** 2017-06-28

**Authors:** Han Tang, Lijie Tan, Yaxing Shen, Hao Wang, Miao Lin, Mingxiang Feng, Songtao Xu, Weigang Guo, Cheng Qian, Tianshu Liu, Zhaochong Zeng, Yingyong Hou, Zhentao Yu, Hongjing Jiang, Zhigang Li, Chun Chen, Changhong Lian, Ming Du, Hecheng Li, Deyao Xie, Jun Yin, Naiqing Zhao, Qun Wang

**Affiliations:** 1 Department of Thoracic Surgery, Zhongshan Hospital, Fudan University, No. 180 Fenglin Road, Xuhui District, Shanghai, 200032 People’s Republic of China; 2 Department of Medical Oncology, Zhongshan Hospital, Fudan University, Shanghai, 200032 China; 3 Department of Radiotherapy, Zhongshan Hospital, Fudan University, Shanghai, 200032 China; 4 Department of Pathology, Zhongshan Hospital, Fudan University, Shanghai, 200032 China; 5 Department of Esophageal Cancer, Tianjin Medical University Cancer Institute and Hospital, Key Laboratory of Cancer Prevention and Therapy of Tianjin City, Tianjin, 300060 China; 6 Department of Thoracic Surgery, Shanghai Chest Hospital, Shanghai Jiaotong University, Shanghai, 200032 China; 7 Department of Thoracic Surgery, Fujian Medical University Fujian Union Hospital, Fuzhou, Fujian 350001 China; 8 Department of General Surgery, Heping Hospital, Changzhi Medical College, Changzhi, Shanxi 046000 China; 9 Department of Cardiothoracic Surgery, The First Affiliated Hospital of Chongqing Medical University, Chongqing, 400016 China; 10 Department of Thoracic Surgery, Ruijin Hospital, Shanghai Jiaotong University School of Medicine, Shanghai, 200025 China; 11 Department of Cardiothoracic Surgery, The First Affiliated Hospital of Wenzhou Medical University, Wenzhou, Zhejiang 325035 China; 12 Department of Cardiothoracic Surgery, Affiliated People’s Hospital of Jiangsu University, Zhenjiang, Jiangsu 212002 China; 13 Department of Biostatistics, School of Public Health, Fudan University, Shanghai, 200032 China

**Keywords:** Esophageal esophageal squamous carcinoma, Neoadjuvant chemoradiation, Neoadjuvant chemotherapy, Minimally invasive esophagectomy

## Abstract

**Background:**

Neoadjuvant chemoradiation is not recommended as an approach for treatment of esophageal squamous cell carcinoma due to its significant postoperative mortality. However, it is assumed the combination of neoadjuvant chemoradiation with minimally invasive esophagectomy (MIE) may reduce postoperative mortality, which can revive preoperative chemoradiation. No randomized controlled studies comparing neoadjuvant chemoradiation plus MIE with neoadjuvant chemotherapy plus MIE have been performed so far. The present trial is initiated to obtain valid information whether neoadjuvant chemoradiation plus MIE yields better survival without worse postoperative morbidity and mortality in the treatment of locally advanced resectable esophageal squamous cell carcinoma(cT_3-4a_N_0-1_M_0_).

**Methods/design:**

CMISG1701 is a multicenter, prospective, randomized, phase III clinical trial, investigating the safety and efficacy of neoadjuvant chemoradiation plus MIE compared with neoadjuvant chemotherapy plus MIE. Patients with locally advanced resectable esophageal squamous cell carcinoma (cT_3-4a_N_0-1_M_0_) are eligible for the study. A total of 264 patients are randomly assigned to neoadjuvant chemoradiation (arm A) or neoadjuvant chemotherapy (arm B) with a 1:1 allocation ratio. The primary outcome is overall survival assessed with a minimum follow-up of 36 months. Secondary outcomes are progression-free survival, recurrence-free survival, postoperative pathologic stage, treatment-related complications, postoperative mortality as well as quality of life.

**Discussion:**

The objective of this trial is to identify the superior protocol with regard to patient survival, treatment morbidity/mortality and quality of life between neoadjuvant chemoradiation plus MIE and neoadjuvant chemotherapy plus MIE.

**Trial registration:**

NCT03001596 (December 17, 2016).

**Electronic supplementary material:**

The online version of this article (doi:10.1186/s12885-017-3446-7) contains supplementary material, which is available to authorized users.

## Background

Esophageal cancer is one of the most common digestive tract cancers worldwide [1]. It is reported the incidence and death rate of esophageal cancer in China is the highest in the world, with its morbidity expecting to ascend to the third place and its mortality expecting to rise to the fourth position according to the Cancer Statistics in China, 2015 [2]. Notably, esophageal squamous cell carcinoma (ESCC) accounts for more than 90% of all cases in China. Traditional curative esophagectomy still plays an important role in the treatment of esophageal cancer, however, curative resection alone often accompanies with high recurrence and metastasis rates, low 3 and 5-year overall survival, especially in patients with locally advanced resectable esophageal cancer(cT_3-4a_N_0-1_M_0_) [1]. Therefore, multimodality therapy has been developed in order to improve the prognosis.

Neoadjuvant therapy has been explored for many years in western countries and Japan, and proved to get survival benefit, especially for locally advanced esophageal cancer. The CROSS trial performed by van Hagen et al. [3] was acknowledged as the most representative one among studies comparing neoadjuvant chemoradiation (nCRT) plus surgery versus surgery alone for patients with adenocarcinoma or squamous cell carcinoma of the esophagus. Patients with esophageal cancer staging as cT_1_N_1_M_0_ or cT_2–3_ N_0-1_ M_0_ were enrolled in the study, and it showed better R0 rate (92% vs 69%, *P* < 0.001), lower node-positive rate (31% vs 75%, *P* < 0.001) and longer overall survival (49.4 vs 24 months, *P* = 0.003) in the nCRT group without significant postoperative morbidities and mortalities. The benefit of nCRT on survival was also confirmed in subgroups with ESCC. Nowadays, many studies [4–7] verified the fact that a significant overall survival benefit was achieved with nCRT plus surgery compared to surgery alone for patients with ESCC. However, accumulating evidence suggested that a significant level of toxicity resulted from nCRT for ESCC. Specifically, FFCD 9901 trial [8] indicated nCRT resulted in significant postoperative mortality (11.1% vs 3.4%, *P* = 0.049) without benefits of 3-year overall survival rate (47.5% vs 53.0%, *P* = 0.94), which was stopped for anticipated futility. In addition, Kumagai et al. [9] summarized 23 RCTs about neoadjuvant therapy via meta-analysis, and it also demonstrated nCRT plus surgery was associated with a significantly higher risk of total postoperative mortality (HR = 1.95, *P* = 0.032) and treatment-related mortality (RR 1·97, *P* = 0·030) compared with surgery alone. Thereafter, nCRT has not been perceived as a safe approach, while neoadjuvant chemotherapy (nCT), which showed an improved survival rate compared with surgery alone, has been demonstrated safe by many studies [6, 9–11] and is being applied as an standard approach for treatment of ESCC.

With the development of techniques and innovation of instruments, minimally invasive esophagectomy (MIE) is introduced into practice worldwide. Due to less trauma, fewer complications as well as similar curative effect, MIE tends to take the place of traditional open esophagectomy and becomes the mainstream procedure [12–14]. There is no doubt that higher postoperative mortality of nCRT results partly from the huge trauma caused by open esophagectomy. Therefore, it is worthwhile to investigate whether MIE could lower the risk of mortality in nCRT approach. Some retrospective studies reported MIE was an acceptable surgical therapy for advanced-stage esophageal malignancies after nCRT without evidence of increased morbidity or mortality [15, 16]. As far as I can see, there are only two prospective randomized studies exploring the outcomes between nCRT plus surgery and nCT plus surgery, which showed higher complete response rate, lower recurrence rate and improved 3-year overall without increased mortality in nCRT group, but it should be pointed that these two studies were confined to esophageal adenocarcinoma [17, 18].

As is known, there are no any studies concentrating on comparing nCRT to nCT followed by MIE in patients with locally advanced resectable ESCC (cT_3-4a_N_0-1_M_0_) so far. Our preliminary work confirmed nCRT followed by MIE was a safe and effective option to treat locally advanced resectable ESCC (cT_3-4a_N_0-1_M_0_) compared with nCT, of which initial results showed higher complete response rate, lower node-positive rate and longer survival time without increased morbidity and mortality (data not published). Hereby, we launch this multicenter prospective randomized phase III clinical trial aiming at investigating and verifying the advantage of nCRT plus MIE in treatment of ESCC, this is the only comparative analysis on nCRT versus nCT in patients with locally advanced resectable ESCC (cT_3-4a_N_0-1_M_0_).

### Rationale

According to the given evidence, a survival benefit of nCRT or nCT plus surgery over surgery alone for locally advanced resectable ESCC has been proved in many RCT studies, but the potential higher risk of postoperative mortality imposes restrictions on nCRT’s application in treating ESCC. As is known to all, MIE has significant advantages in decreasing postoperative morbidity and mortality compared with open surgery and has been proved to be feasible in nCRT, however, we have no idea about whether it could reduce mortality when combined with neoadjuvant therapy. As neoadjuvant therapy plus MIE is extensively and successfully applied in clinical practice in patients with ESCC and no RCTs have concentrated on comparing the outcomes between nCRT and nCT followed by MIE, there is a clear need to obtain evidence concerning the value of nCRT plus MIE in patients with locally advanced resectable ESCC (cT_3-4a_N_0-1_M_0_) from a multicenter RCT.

## Methods/design

### Study design

**Fig. 1:**
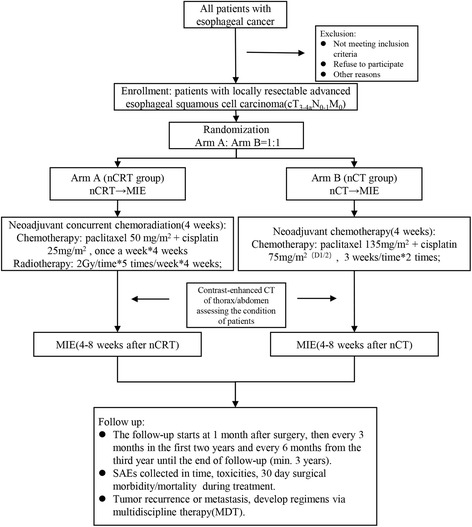
Trial diagram

CMISG1701 is a two-arm randomized phase III trial in which every patient is randomly assigned to nCRT (arm A) or nCT (arm B) with a 1:1 allocation ratio (Fig. 1). The objective of this trial is to investigate the safety and efficacy of nCRT plus MIE procedure in treating ESCC. To achieve this goal, 364 patients will be recruited from 9 participating medical center across China. Written informed consent is obtained from all patients prior to participation in the trial. All participating centers are highly experienced in MIE and perform at least 40 combined modality therapies of patients with localized ESCC per year. Additionally, blood and tissue samples will be collected for translational research.

### Target population

Patients of both genders with thoracic ESCC will be considered to be enrolled in the trial. Responsible investigators evaluate the conditions of patients and ensure meeting the selection criteria. Besides, only patients providing written informed consent are allowed to enter the trial.

### Sample size considerations

The sample size calculations are based on the primary outcome overall survival. From our own experience, the 3-year overall survival rate is 72.7% and 47.1% for patients in nCRT group and in nCT group without differences in mortality, respectively (data not published). Therefore, the total number of sample size is 264, which is based on the intention of showing a benefit of nCRT arm (arm A) over the other arm (arm B) in the primary end point of 20% with a one-sided type I error of 5% and a power of 90% as well as 15% drop out before surgery or lost to follow up according to Power Analysis and Sample Size (PASS). Thus, 134 patients were enrolled in each arm with the balance of age, N stage and trial center according to 1:1 randomized allocation. The sample size will ensure sufficient power to demonstrate an overall survival advantage of nCRT over nCT by the end of the trial.

### Inclusion criteria

Eligible patients must meet all of the following criteria:

1. Histologically-confirmed squamous cell carcinoma of the esophagus;
2. Tumors of the esophagus are located in the thoracic cavity;
3. Pre-treatment stage as cT_3-4a_N_0-1_M_0_ (AJCC/UICC 7th Edition) (In case of stage cT_4a_, curative resectability has to be explicitly verified by the local surgical investigator prior to randomization).
4. Age is between 18 years and 75 years,
5. Eastern Cooperative Oncology Group (ECOG) performance status 0–1;
6. Adequate cardiac function. All patients should perform ECG, and those with a cardiac history or ECG abnormality should perform echocardiography with the left ventricular ejection fraction >50%.
7. Adequate respiratory function with FEV1 ≥ 1.2 L, FEV1% ≥ 50% and DLCO ≥ 50% shown in pulmonary function tests.
8. Adequate bone marrow function (White Blood Cells >4 × 10^9 /L; Neutrophil >2.0 × 10^9 /L; Hemoglobin >90 g/L; platelets > 100 × 10^9 /L);
9. Adequate liver function (Total bilirubin <1.5× Upper Level of Normal (ULN); Aspartate transaminase(AST) and Alanine transaminase (ALT) <1.5× ULN);
10. Adequate renal function (Glomerular filtration rate (CCr) >60 ml/min; serum creatinine (SCr) ≤120 μmol/L);
11. The patient has provided written informed consent and is able to understand and comply with the study;

### Exclusion criteria

Patients meeting any of the following criteria are not eligible for this trial:

1. Patients with non-squamous cell carcinoma histology;
2. Patients with advanced inoperable or metastatic esophageal cancer;
3. Pre-treatment stage as cT_1-2_ N_0-1_ M_0_ (AJCC/UICC 7th Edition);
4. Pre-treatment stage as cN_2–3_ or cT_4b_ (non-curatively-resectable verified by the local surgical investigator, AJCC/UICC 7th Edition);
5. Patients with another previous or current malignant disease which is likely to interfere with treatment or the assessment of response in the judgement of the local surgical investigator.
6. Any patient with a significant medical condition which is thought unlikely to tolerate the therapies. Such as cardiac disease (e.g. symptomatic coronary artery disease or myocardial infarction within last 12 months), clinically-significant lung disease, clinically-significant bone marrow, liver, renal function disorder;
7. Pregnant or lactating women and fertile women who will not be using contraception during the trial;
8. Allergy to any drugs;
9. Participation in another intervention clinical trial with interference to the chemotherapeutic or chemoradiotherapeutic intervention during this study or during the last 30 days prior to informed consent;
10. Expected lack of compliance with the protocol.

### Study interventions

#### Neoadjuvant chemoradiation (arm A)

The nCRT arm consists of a combination of preoperative radiotherapy and chemotherapy. The patient receives 4 weeks of radiation therapy and concurrent weekly cycles of chemotherapy. The detailed scheme runs as follows: radiotherapy with 40Gy is delivered in 20 fractions of 2Gy: days 1–5, days 8–12, days 15–19 and days 22–26; Chemotherapy: paclitaxel 50 mg/m^2^ day 6, 13, 20, 27 and cisplatin 25 mg/m^2^ day 6, 13, 20, 27 at the intervals of radiotherapy.

#### Neoadjuvant chemotherapy (Arm B)

The nCT arm consists of two cycles of preoperative chemotheraoy before surgery. The regimen is composed of paclitaxel and cisplatin. The detailed scheme is listed as follows: paclitaxel 135 mg/m^2^ on day 1 and cisplatin 75 mg/m^2^ on day 1 by intravenous drip infusion, which is given the second cycle after 3 weeks.

#### Minimally invasive esophagectomy (Both arms)

After 4–8 weeks of neoadjuvant therapy, MIE will be performed. The procedure in details is referred in previous article [19–21]. To achieve an accurate ypTNM stage, the extent of lymphadenectomy demands resecting radically. Dissected lymph nodes were classified according to lymph node stations adopted by the Japanese Classification [22]. The dissected nodes in thoracic cavity should include the upper paraesophageal (no.105), paratracheal (no.106r and 106tb), subcarinal (no.107), middle paraesophageal (no.108), bilateral hilar lymph nodes (no.109), lower paraesophageal (no.110), posterior mediastinal lymph nodes (no.111), and diaphragmatic (no.112) ones. The dissected abdominal nodes should include the nodes lateral to the paracardia, lesser curvature, greater curvature, left gastric, common hepatic, splenic, and celiac stations. If neoplasm is located at upper mediastinum, cervical nodes in the cervical paraesophageal (no.101) and supraclavicular regions (no.104) should be dissected.

### Outcome measures

#### Primary outcome

The primary outcome is the overall survival time in the intent-to-treat population, which ends with the date of death of any causes since the date of randomization assessed up to 36 months. For patients alive at study closure, the survival time will be censored at time of last known survival status.

#### Secondary outcomes

1. Progression-free survival (PFS) time: It is defined as the time from the date of randomization to the date of first recurrence/progression (local, regional or distant) or death assessed up to 36 months. Progression is examined by computed tomography (CT), positron emission tomography-computed tomography (PET-CT) and/or upper endoscopy.
2. Recurrence-free survival (RFS) time: It is defined as the time from the date of surgery to the date of first recurrence (local, regional or distant) or death assessed up to 36 months. Recurrence is examined by CT, PET-CT and/or upper endoscopy.
3. Postoperative pathologic stage:

- ♦ Pathological complete response rate(pCR): Pathological complete response rate (pCR) is to be assessed in the resected specimen following neoadjuvant therapy using standardized work up of the resection specimen in the pathology department and standardized histological criteria for tumor regression grading. The degree of histomorphologic regression is clarified into four categories as follows: grade 1, no evidence of vital residual tumor cells (pathological complete response); grade 2, less than 10% vital residual tumor cells; grade 3, 10 to 50%; and grade 4, more than 50% according to previous report [23].
- ♦ R0 resection rate: No vital tumor is presented at the proximal, distal, or circumferential resection margin, then it is considered to be R0 resection. If a vital tumor is shown at 1 mm or less from the proximal, distal, or circumferential resection margin, it is considered to be microscopically positive (R1).
- ♦ Positive lymph nodes’ number: According to pathological reports, record the number of positive lymph nodes.
- ♦ Postoperative TNM stage according to the UICC TNM7 system [24].

4. Treatment related complications: Record the data according to International Consensus of Esophagectomy Complications Consensus Group (ECCG) [25]. Chemoradiation/chemotherapy-related toxicities during preoperative time are collected according to CTCAE version 4.03;
5. Postoperative mortality: 30-day postoperative mortality;
6. Quality of life(QOL): QOL is respectively evaluated at randomization, 4 weeks after neoadjuvant therapy and 1 month, 4 month, 7 month and yearly after surgery among patients by using the European Organization for Research and Treatment of Cancer Quality of Life Questionnaire C-30 (EORTC QLQ-C30) and EORTC QLQ-OES18, it is assessed up to 36 months.

### Assessments, data collections and follow-up

#### Pre-therapeutic assessments

Routine examination is performed and recorded for every potential patient prior to any treatment, which includes a physical examination, demography, medical history, vital signs, body weight, electrocardiogram (ECG), standard laboratory tests (preoperative items), upper endoscopy with biopsies, upper gastrointestinal contrast, pulmonary function, endoscopic ultrasound of the neck and a CT scan of the thorax and abdomen (It could be replaced by PET/CT, if an agreement reaches between doctor and patient). If the patient has a history or symptom of cardiac diseases, cardiac ultrasound is given. All items above should be finished within 2 weeks before surgery. The inclusion and exclusion criteria are checked and validated with these examinations.

#### Assessments during the treatment phase

Vital sign, body weight, description of discomfort symptoms and standard laboratory tests (blood routine, blood biochemistry) are obtained and recorded weekly during neoadjuvant therapy period, which assesses the toxicity of preoperative therapy. After 4–6 weeks of neoadjuvant therapy, a CT (PET-CT) scan of thorax and abdomen and ultrasound of the neck to re-stage of the tumor and quality of life questionnaires to evaluate patients’ conditions are performed. On the day of discharge from hospital after surgery, standard laboratory tests, body weight, surgery-related data, pathological report as well as post-operative data are assessed.

#### Assessments during the follow-up phase

The first follow-up visit is performed 1 month after surgery. From then on, follow-up visits are carried out every 3 months in the first 2 years of follow-up and every 6 months from the third year until the end of follow-up (min. 3 years). For all patients, follow-up assessment is performed until the end of the trial or death. The end of the trial will be 3 years after the study treatment of the last patient started. The detailed examination items include standard laboratory tests (blood routine, tumor biomarker), a CT scan of thorax, an ultrasound of the neck and abdomen and quality of life questionnaires (EORTC C-30 + OES-18).

#### Translational research

The clinical trial includes tissue samples and blood samples collection for future translational research and the development/validation of biomarkers. Trial participants will be asked for additional optional consent to participate in this aspect of the study. The standard tissue sample consists of preoperative biopsy tumor tissue, biopsy normal mucosa, and postoperative tumor tissue, normal mucosa. The blood samples include 2 × 5 ml EDTA blood samples taken at the time of pre-treatment, pre-surgery, 4 month after surgery and recurrence, respectively. All samples will be stored at the Zhongshan Hospital Cancer Bank for future translational researches.

Detailed information on all examinations, assessments during the screening phase, treatment phase and follow-up phase are given in Additional file 1: Table S1.

### Statistical analysis

Data are analyzed according to the intention-to-treat principle in all randomized patients. Comparisons between the groups will be done with the χ^2^ test and Fisher’s exact test for categorical parameters, while with Student’s t test or analysis of variance (ANOVA) test for continuous variables. Survival rates in the two treatment arms will be estimated by the Kaplan-Meier method. Then, the Cox proportional hazard model and the log rank test will be used to evaluate the survival-independent factors. The significance level is set at 0.05.

### Funding and current status

CMISG1701 has been ethically approved by the ethics committee of the Zhongshan Hospital (B2016-177R). The study is supported by Foundation of Science and Technology Commission of Shanghai Municipality (16411965900) and Clinical Research Foundation of Zhongshan Hospital (2016ZSLC15). The study protocol has also undergone peer-review by these two government funding body.

Our study began to recruit in January 1st, 2017. It is still at the stage of recruiting as 40 patients have been recruited since March 3rd, 2017.

## Discussion

Great success in improving prognosis of patients with esophageal cancer has been achieved in the past 20 years due to a persistent effort. In addition to diagnosis of early stage, perioperative critical care and surgical technique, multimodality therapy plays a great role [5]. In recent years, neoadjuvant therapy for esophageal cancer gains popularity around the world due to a series of RCTs [6, 9, 10], especially the CROSS trial [3, 26], which is awarded as the top-10 medicine progress in 2012. All these trials indicated nCRT had great significances in improving R0 rate, lowering the TNM stage and prolonging the survival time in ESCC. However, accumulating evidence [8, 9] suggested a significant level of toxicity resulted from neoadjuvant chemoradiation for ESCC, which hindered its implementation. Compared to surgery alone, nCT has its advantage in efficacy without increasing mortality, therefore, nCT is perceived as the standard therapeutic procedure for locally advanced resectable ESCC. As is known to all, MIE has great superiorities in reducing trauma [12], and a great deal of work has been done to promote the spread of MIE in China in recent years, consequently, MIE gains its popularity in most of the regional medical centers, which is proved to be safe in patients receiving neoadjuvant therapy. Therefore, the issue whether the combination of nCRT plus MIE could lower the risk of postoperative mortality for treatment of locally advanced resectable ESCC is interesting and worth investigating. Although different studies have been carried out comparing either nCRT or nCT plus surgery versus surgery alone for ESCC, no prospective data comparing the contemporary regimens of nCRT and nCT in patients with locally advanced resectable ESCC are available, let alone the involvement of MIE. Nowadays, nCT is still the most widely-established treatment modality for esophageal cancer in China. As nCT has already been proven by RCTs [9–11] to be superior to surgery alone, it serves as the control group and no surgery alone group is added. The regimen of nCRT plus MIE has been used frequently in specialized Chinese medicine centers and shows promising outcomes in retrospective analysis.

Based on the above, there is a growing clinical consensus that the two strategies of neoadjuvant therapy plus MIE should be compared head-to-head in a prospective randomized controlled trial in locally advanced resectable ESCC (cT_3-4a_N_0-1_M_0_). This trial aims to obtain valid information whether nCRT plus MIE yields superior benefits for the curative treatment of ESCC.

## Conclusion

CMISG1701 is a multicenter, two-arm randomized phase III trial, comparing neoadjuvant chemoradiation plus MIE with neoadjuvant chemotherapy plus MIE for the treatment of locally advanced resectable esophageal squamous cell carcinoma(cT_3-4a_N_0-1_M_0_). It is estimated neoadjuvant chemoradiation plus MIE has advantages in improving survival time without increasing mortality.

## Additional files

Additional file 1: Table S1. Treatment schedule. (DOCX 20 kb)
