# Supplementary material for: CMISG1701: a multicenter prospective randomized phase III clinical trial comparing neoadjuvant chemoradiotherapy to neoadjuvant chemotherapy followed by minimally invasive esophagectomy in patients with locally advanced resectable esophageal squamous cell carcinoma (cT3-4aN0-1M0) (NCT03001596)
Source: BMC Cancer. 2017 Jun 28;17:450. doi: 10.1186/s12885-017-3446-7 (PMC5490174; doi:10.1186/s12885-017-3446-7)
Supplement: Supplementary file 1 — Treatment schedule. (DOCX 20 kb) [file 12885_2017_3446_MOESM1_ESM.docx]

**Treatment Schedule**

| **Treatment Phase** | **Screening** | **Neoadjuvant treatment** | **Preoperative evaluation** | **Operation** | **Follow up** |
| --- | --- | --- | --- | --- | --- |
| Time point | <14 days before Randomization | Week 1-5  At day 1 of each week | Within 3-5 weeks after neoadjuvant treatment | Day of hospital  discharge from Surgery | Starting 1 month after surgery, every  3/6 months^[[1]](#endnote-1)^* |
| Items | **(Vs)** | **(V_n_)** | **(Vr)** | **(Vs)** | **(F1--x)** |
| Informed consent | x |  |  |  |  |
| Inclusion/exclusion | x |  |  |  |  |
| Demography^[[2]](#endnote-2)^ | x |  |  |  |  |
| Medical history | x |  |  |  |  |
| Vital sign(P, R, T, BP) | x | x | x | x |  |
| Physical examination^[[3]](#endnote-3)^ | x |  | x |  | x |
| Body weight | x | x | x | x | x |
| ECOG performance score | x |  |  |  |  |
| CT thorax/abdominal | x^[[4]](#endnote-4)^ |  | x |  | x^[[5]](#endnote-5)^ |
| Endoscopic ultrasound | x |  | x |  | x |
| Upper GI endoscopy | x |  |  |  |  |
| gastroscopy | x |  |  |  | x^[[6]](#endnote-6)^ |
| Histopathology report | x |  |  | x |  |
| Tissue specimen^[[7]](#endnote-7)^ | x |  |  | x |  |
| ECG^[[8]](#endnote-8)^ | x |  | x |  |  |
| Pulmonary function | x |  | x |  |  |
| Blood routine and biochemistry ^[[9]](#endnote-9)^ | x | x | x | x |  |
| Laboratory Infection^[[10]](#endnote-10)^ | x |  |  |  |  |
| Laboratory Coagulation^[[11]](#endnote-11)^ | x |  | x |  |  |
| Tumor biomarker^[[12]](#endnote-12)^ | x |  | x |  | x |
| Pregnancy test (only women) | x |  |  |  |  |
| Randomization | x |  |  |  |  |
| Blood sample^[[13]](#endnote-13)^ | x |  |  | x | × |
| Adverse events and complications ^[[14]](#endnote-14)^ | x | | | | |
| Concomitant medication^[[15]](#endnote-15)^ | x | | | | |
| Quality of life (EORTC QLQ-C30, OES18,)^[[16]](#endnote-16)^ | x |  | x | x | x |

1. * The first follow-up visit is performed 1 months after surgery. From then on, follow-up visits are carried out every 3 months (+/- 7days) in the first two years of follow-up and every 6 months (+/- 7days) from the third year after treatment until the end of follow-up (min. 3 years). [↑](#endnote-ref-1)
2. Demography includes sex, age, height, race, ethnicity, job category, allergy and so on [↑](#endnote-ref-2)
3. Physical examination includes, but is not limited to, cardiovascular, gastrointestinal, hepatobiliary, respiratory, musculoskeletal, skin, neurological, genitourinary/renal and other organ systems. [↑](#endnote-ref-3)
4. Not older than 14 days before date of randomisation，and it must be contrast-enhanced CT. If suspected to be T_4b_ stage, multiregional lymph node metastases or distant metastases, PET-CT or endoscopic ultrasound (EUS) (selectable) is performed to ensure pre-treatment cTNM stage. [↑](#endnote-ref-4)
5. Contrast-enhanced CT of Thorax/Abdomen is carried out regularly at follow-up visits. Further diagnostic investigations, including PET-CT, Upper endoscopy are performed only if suspected to be recurrence or metastasis at the discretion of the investigator/treating physician. [↑](#endnote-ref-5)
6. Gastroscopy is performed once a year. [↑](#endnote-ref-6)
7. Representative blocks from the initial biopsy and the operative specimen will be requested from the reporting pathologists. [↑](#endnote-ref-7)
8. ECG must be performed during screening and preoperative. Patients with a cardiac history should have echocardiography, further cardiac examinations can be performed if necessary to exclude contraindication. [↑](#endnote-ref-8)
9. Blood routine includes hemoglobin, total red count, total white blood count, platelet count, and a differential white count including neutrophils, lymphocytes, monocytes, eosinophils and basophils. Biochemistry includes (but is not limited to) AST, ALT, total bilirubin, blood glucose, serum creatinine, sodium, potassium. [↑](#endnote-ref-9)
10. HBV、HCV、HIV serological examinations [↑](#endnote-ref-10)
11. Coagulation includes PT, PTT和INR。 [↑](#endnote-ref-11)
12. Tumor biomarker includes CEA, CA19-9, CA125, CYFRA21-1 and SCC. [↑](#endnote-ref-12)
13. Two blood samples are collected for translational research before treatment, before surgery, 4 months after surgery and the time of recurrence or metastasis, respectively. [↑](#endnote-ref-13)
14. The AE reporting period for this trial begins after first intake of medication within the study and until 8 months after randomisation. All adverse events have to be documented in the CRF. [↑](#endnote-ref-14)
15. Concomitant medication must be available in the source data and don`t be captured in the CRF. [↑](#endnote-ref-15)
16. Quality of life is recorded before treatment, 4 weeks after neoadjuvant therapy and 1 month, 4 month, 7 month, yearly after surgery, respectively. [↑](#endnote-ref-16)
